# Position and ranking influence in padel: somatic anxiety and self-confidence increase in competition for left-side and higher-ranked players when compared to pressure training

**DOI:** 10.3389/fpsyg.2024.1393963

**Published:** 2024-04-04

**Authors:** Rafael Conde-Ripoll, Adrián Escudero-Tena, Álvaro Bustamante-Sánchez

**Affiliations:** Faculty of Sport Sciences, Universidad Europea de Madrid, Madrid, Spain

**Keywords:** psychology, confidence, anxiety, competition, practice, padel, CSAI-2R, STAI-S

## Abstract

This study aimed to analyze the differences in the precompetitive anxiety and self-confidence according to the side of play, the ranking and the match outcome, under different competitive scenarios, in high level men’s padel players from Finland who trained under pressure prior to the competition. 10 men’s padel players (28.60 (4.17) years old) from the highest category participated in the research. The CSAI-2R (Competitive State Anxiety Inventory-2 Revised) and STAI-S (State–Trait Anxiety Inventory – State) questionnaires were used and descriptive and inferential analyzes were performed, including Mann–Whitney’s *U* tests. The findings illuminate that, across the player spectrum, somatic anxiety and self-confidence levels are higher before competition compared to training matches. This trend holds true for left-side, higher-ranked and match winning players. Even lower-ranked players exhibit heightened self-confidence preceding competitions. These insights offer valuable considerations for players, coaches, and sports psychologists, fostering a deeper understanding of the intricate interplay between pressure training, competition, and the athlete’s psychological landscape.

## Introduction

The global surge of padel, a doubles racket sport, has attracted participants from over 60 nations affiliated with the International Padel Federation (2024). This growth has triggered a notable rise in scholarly research focused on psychophysiology (Conde-Ripoll et al., 2023; Díaz-García et al., 2023a; Bustamante-Sánchez et al., 2024), although technical-tactical performance remains a dominant area of study (Lupo et al., 2018; Escudero-Tena et al., 2021; Martín-Miguel et al., 2023; Conde-Ripoll et al., 2024; Ungureanu et al., 2024).

Participating in sports may involve rigorous physical exertion and significant psychological burdens, posing challenges for certain athletes to handle and potentially resulting in adverse outcomes (Balaguer et al., 2012). This phenomenon becomes more pronounced when high-level padel players are required to compete in two consecutive matches within a single day, leading to a notable build-up of cognitive exhaustion which intensifies perceived mental fatigue (Díaz-García et al., 2023b). On its part, anxiety encompasses a negative emotional state characterized by nervousness, worry, and apprehension, typically accompanied by heightened physiological arousal (Weinberg and Gould, 2010). This state leads to an individual’s diminished adaptability across physiological, behavioral, and cognitive realms, thereby obstructing athletic performance (Tamorri, 2004). Anxiety often escalates immediately before competition and subsides shortly afterward (Gould et al., 1984). Numerous studies have confirmed that heightened pre-competitive anxiety can hinder athletic performance (Burton, 1988).

To comprehend this anxious state, the multidimensional theory (Martens et al., 1990) suggests that subjective expressions of anxiety involve cognitive and somatic components, along with self-confidence. The somatic aspect encompasses the physiological and emotional facets of anxiety, originating directly from organismic activation (Martens et al., 1990). It encompasses a range of physical symptoms (elevated levels of physiological function activation) triggered automatically by the demands of competition (Dosil, 2004), often accompanied by nervousness and tension. Conversely, cognitive anxiety pertains to the mental dimension of anxiety, arising from negative expectations or low self-confidence levels in oneself and one’s abilities (Martens et al., 1990). A third aspect, self-confidence, while not a direct measure of anxiety, can influence athletes’ experience of cognitive anxiety. Self-confidence reflects an individual’s belief in their capacity to manage their surroundings and themselves (Woodman and Hardy, 2001). Prior to Spanish competitions, high-level (first category) padel players exhibit higher levels of self-confidence and lower levels of somatic-anxiety when compared to players from third category (Castillo-Rodriguez et al., 2022).

Training should be tailored to prepare players for competition in the best possible way. A comparison between the demands of competition and training suggests that the former typically imposes higher physiological demands, coupled with psychological factors such as the allure of victory, competition prestige, and the challenge posed by rival athletes. Souza et al. (2019) observed elevated somatic anxiety, LH/HF ratio, and cortisol levels preceding competitions in contrast to training, what suggest an excitement of the autonomous nervous system on its sympathetic division. As relevant competitions approach, as exemplified in our study with training occurring 2 weeks prior to a key event, research suggests an increase in stress and anxiety levels among athletes (Loupos et al., 2008; Morales et al., 2014; Bustamante-Sánchez et al., 2024). In racket sports, contemporary methodologies advocate for competitive training scenarios mirroring match-play conditions to enhance decision-making proficiency in authentic settings (Mecheri et al., 2019; Navia et al., 2022). It is important to highlight that in racket sports like padel, where rapid decision-making is constant (Castillo-Rodríguez et al., 2014), effectively managing pressure situations directly influences performance (González-Díaz et al., 2012; Knight et al., 2016; Martínez-Gallego et al., 2022). Since mental strength plays a pivotal role in sports success (Crust and Keegan, 2010) and in upholding or boosting competitive performance (Gucciardi et al., 2015), players need to develop their mental skills (Mamassis and Doganis, 2004) as well as pressure should be present during training sessions (Low et al., 2021). Although replicating competition can be difficult, training under mild levels of pressure can still benefit future performance under higher levels of pressure (Oudejans and Pijpers, 2010). Pressure training is an intervention that applies pressure on athletes while they practice their sport with the aim of helping them improve their performance under pressure (Low et al., 2021). In other words, it attempts to increase pressure above the level that athletes feel in a typical training session. In this regard, Stoker et al. (2016) examined stressors that elite-level coaches, among a wide spectrum of sports like badminton and table tennis, used to intentionally create pressure during training sessions. The ensuring framework categorized stressors into demands and consequences. The former increased the difficulty to perform (for example, adding distractions to the environment or changing the rules of a drill), whereas the latter included rewards (e.g., the change to choose the next tournament abroad), forfeits (e.g., having to miss a training session), or judgment (e.g., being watched by the professional team’s performance director). Subsequent research found that consequences increase pressure more than demands do (Stoker et al., 2017, 2019).

Despite the growing interest in padel, a notable gap exists in the scientific literature regarding the consideration of pressure training, the comparison of precompetitive anxiety levels between training and competition contexts, and between players based on their side of play. Our study aims to address this gap by investigating these aspects within the padel domain, providing valuable insights into the psychological dynamics of player performance. By examining the effects of pressure training and elucidating differences in anxiety levels between training and competition, and between right- and left-side players, we aim to offer a novel contribution to the field. These findings hold significant practical implications for players, coaches, and sports psychologists, as they can inform tailored strategies for optimizing player performance and well-being. Players may benefit from lifestyle modifications and adjustments to playing style, while coaches and psychologists can tailor training sessions and feedback to better support players in managing anxiety and enhancing self-confidence.

Therefore, the aim of the present investigation was to analyze the differences in the pre-competitive anxiety and self-confidence between training and competition in high-level men’s padel players from Finland who trained under pressure, according to the side of play, the ranking, and the match outcome. The following hypotheses were put forward: (1) prior to competition, athletes will show higher levels of anxiety and lower levels of self-confidence than prior to training matches, (2) left-side players, as well as right-side players, will show higher levels of self-confidence and less anxiety before competition than prior to training matches, (3) both prior to training and competitive matches, left-side players will present lower levels of anxiety and higher levels of self-confidence when compared to right-side players, (4) higher ranked players will show similar levels of anxiety and self-confidence between competition and training matches while lower ranked players will show lower levels of self-confidence and higher levels of anxiety before competition than training matches, (5) match winning players will show similar levels of anxiety and self-confidence between competition and training matches while match losing players will show lower levels of self-confidence and higher levels of anxiety before competition than training matches.

## Materials and methods

### Sample and participants

A total of 10 men’s high level padel players (28.60 (4.17) years old) from Finland voluntarily participated in the present study. All participants were ranked top 25 in Finland. None of the athletes had physical injuries, nor were they using any medication. In addition, none of the participants had any reason that prevented them from participating in the study. The sample consisted of 20 matches (training: 11, competition: 9). The training matches took place at Padel Tampere Linnakallio New during the 2 weeks prior to the 2023 Finnish national championship by pairs. The obtained points of this competition counted for the ranking of the Finnish Federation. The study was in accordance with the Helsinki Declaration (World Medical Association, 2013). Participants were treated ethically under the American Psychological Association code of ethics regarding consent, anonymity and responses. Previously, the current investigation had been approved by the Ethics Committee of the European University of Madrid with the code CIPI/22.303. So as to respect the principles of voluntariness and confidentiality, each player was required to sign an informed consent form that clearly explained the objectives of the research and their voluntary participation in it. To obtain permission to administer the questionnaires to the players before the competition, the researchers first contacted the Finnish Padel Federation and the championship organizer.

### Instruments

#### Competitive anxiety

CSAI-2R was used to measure precompetitive anxiety and self-confidence of players (Cox et al., 2003) and STAI-S was used to measure their state anxiety (Spielberger et al., 1970). These questionnaires have been used in previous research in padel (Conde-Ripoll et al., 2023). In the analysis of the CSAI-2R instrument, Cronbach’s alpha coefficients were obtained, showing reliability scores of 0.515 for cognitive anxiety, 0.808 for somatic anxiety, 0.758 for self-confidence, all but the former meeting acceptable standards (Nunnally and Bernstein, 1994; DeVellis, 2003; Vaske, 2008).

### Procedure

The players were informed by the coach that they would undergo pressure training. Pressure training refers to an intervention designed to assist athletes in performing under pressure by deliberately exposing them to stressors during training sessions (Bell et al., 2013; Driskell et al., 2014; Stoker et al., 2016). In our study, players were recorded during the training matches while their technical-tactical performance was exhaustively evaluated by the head coach of the first-ever professional padel team in Finland. Players were informed about this process before the training match began.

The questionnaires were administered to the players between 30 and 45 min prior to the start of each match, following the same criteria to that used by Andrade-Fernández et al. (2007) and Conde-Ripoll et al. (2023). All questionnaires were completed in a quiet room with controlled temperature of 20°C. Participants completed the questionnaires in English, as it is the only language that both researchers and athletes are fluent in. Participants were not allowed to speak during the assessments.

### Statistical analysis

A Kolgomorov-Smirnov test was used to test the normality of the distribution of the data and it indicated that it is non-parametric. Then, results were shown as median and interquartile range.

Next, inferential analyzes were conducted, including Mann–Whitney’s U tests. Additionally, effect sizes (Cohen’s d) were calculated and can be interpreted as small (0.20 to 0.49), moderate (0.50 to 0.79) and large (d ≥ 0.80) (Cohen, 1988).

All data were analyzed using the statistical package SPSS for Macintosh v.25.0 (SPSS Inc., Chicago, IL, United States). A *p* value of less than 0.05 was considered to be statistically significant.

## Results

Results are shown as mean and interquartile range.

Table 1 presents the level of precompetitive anxiety and self-confidence between players at training and at competition matches. Players significantly present higher levels of somatic anxiety (*p* = 0.025; *d* = 0.602) and self-confidence (*p* = 0.002; *d* = 0.848) in competition when compared to training matches.

**Table 1:** Precompetitive anxiety and self-confidence between players at training and at competition matches.

	Training	Competition		
Variable	Median (IQR)	Median (IQR)	*p*	*d*
CA	1.40 (0.40)	1.20 (0.40)	0.371	0.228
SA	1.43 (0.71)	1.71 (0.57)	0.025*	0.602
SC	3.10 (0.60)	3.40 (0.60)	0.002*	0.848
STA	7.00 (5.00)	6.00 (2.00)	0.900	0.032

CA, cognitive anxiety; SA, somatic anxiety; SC, self-confidence; STA, state anxiety; n, number; IQR, interquartile range; *p, p*-value; *p < 0.05; *d*, Cohen’s d.

Table 2 further examines the level of precompetitive anxiety and self-confidence between players at training and at competition matches as a function of the side of play. Left-side players significantly present higher levels of somatic anxiety (*p* = 0.045; *d* = 0.708) and self-confidence (*p* = 0.002; *d* = 1.265) in competition when compared to training matches.

**Table 2:** Precompetitive anxiety and self-confidence between players at training and at competition matches according to the side of play.

	Left-side player				Right-side player			
	Training	Competition			Training	Competition		
Variable	Median (IQR)	Median (IQR)	*p*	*d*	Median (IQR)	Median (IQR)	*p*	*d*
CA	1.40 (0.40)	1.20 (0.10)	0.231	0.404	1.50 (0.70)	1.20 (0.40)	0.801	0.099
SA	1.14 (0.61)	1.57 (0.43)	0.045*	0.708	1.57 (0.57)	1.86 (0.14)	0.201	0.520
SC	3.20 (0.40)	3.40 (0.60)	0.002*	1.265	3.00 (0.70)	3.20 (0.80)	0.175	0.557
STA	5.00 (4.00)	6.00 (2.00)	0.413	0.281	9.50 (3.00)	7.00 (5.00)	0.167	0.569

CA, cognitive anxiety; SA, somatic anxiety; SC, self-confidence; STA, state anxiety; n, number; IQR, interquartile range; *p*, *p*-value; *p < 0.05; *d*, Cohen’s d.

Table 3 depicts the level of precompetitive anxiety and self-confidence between right- and left-side players at training and at competition matches. Before training matches, right-side players present significantly higher levels of somatic anxiety (*p* = 0.016; *d* = 0.873) and state anxiety (*p* < 0.001; *d* = 1.480) than left-side players.

**Table 3:** Precompetitive anxiety and self-confidence between right- and left-side players at training and at competition matches.

	Training				Competition			
	Left	Right			Left	Right		
Variable	Median (IQR)	Median (IQR)	*p*	*d*	Median (IQR)	Median (IQR)	*p*	*d*
CA	1.40 (0.40)	1.50 (0.70)	0.619	0.168	1.20 (0.10)	1.20 (0.40)	0.525	0.285
SA	1.14 (0.61)	1.57 (0.57)	0.016*	0.873	1.57 (0.43)	1.86 (0.14)	0.091	0.754
SC	3.20 (0.40)	3.00 (0.70)	0.281	0.374	3.40 (0.60)	3.20 (0.80)	0.134	0.662
STA	5.00 (4.00)	9.50 (3.00)	< 0.001*	1.480	6.00 (2.00)	7.00 (5.00)	0.260	0.489

CA, cognitive anxiety; SA, somatic anxiety; SC, self-confidence; STA, state anxiety; n, number; IQR, interquartile range; *p*, *p*-value; *p < 0.05; *d*, Cohen’s d.

Table 4 delves into the variation of the levels of precompetitive anxiety and self-confidence between players at training and at competition matches as a function of the ranking. Higher-ranked players present significantly higher levels of somatic anxiety (*p* = 0.005; *d* = 1.075) and self-confidence (*p* = 0.046; *d* = 0.717) at competition when compared to training matches. Lower-ranked players present significantly higher levels of self-confidence (*p* = 0.019; *d* = 1.053) at competition when compared to training matches.

**Table 4:** Precompetitive anxiety and self-confidence between players at training and at competition matches according to the ranking.

	Higher-ranked				Lower-ranked			
	Training	Competition			Training	Competition		
Variable	Median (IQR)	Median (IQR)	*p*	*d*	Median (IQR)	Median (IQR)	*p*	*d*
CA	1.30 (0.40)	1.20 (0.20)	0.397	0.281	1.40 (0.50)	1.20 (0.80)	0.806	0.097
SA	1.29 (0.61)	1.71 (0.50)	0.005*	1.075	1.50 (0.61)	1.71 (0.57)	0.582	0.219
SC	3.20 (0.30)	3.40 (0.80)	0.046*	0.717	3.00 (0.60)	3.40 (0.80)	0.019*	1.053
STA	6.50 (4.00)	6.00 (2.00)	0.767	0.101	8.00 (6.00)	7.00 (5.00)	0.927	0.036

CA, cognitive anxiety; SA, somatic anxiety; SC, self-confidence; STA, state anxiety; n, number; IQR, interquartile range; *p*, *p*-value; *p < 0.05; *d*, Cohen’s d.

Table 5 shows the level of precompetitive anxiety and self-confidence between players at training and at competition matches as a function of the match outcome. Winning players present significantly higher levels of somatic anxiety (*p* = 0.026; *d* = 0.824) and self-confidence (*p* = 0.014; *d* = 0.922) at competition when compared to training matches.

**Table 5:** Precompetitive anxiety and self-confidence between players at training and at competition matches according to the match outcome.

	Winning players				Losing players			
	Training	Competition			Training	Competition		
Variable	Median (IQR)	Median (IQR)	*p*	*d*	Median (IQR)	Median (IQR)	*p*	*d*
CA	1.20 (0.50)	1.20 (0.20)	0.638	0.158	1.40 (0.50)	1.20 (0.80)	0.865	0.065
SA	1.36 (0.61)	1.71 (0.57)	0.026*	0.824	1.43 (0.61)	1.79 (0.36)	0.169	0.554
SC	3.20 (0.40)	3.40 (0.60)	0.014*	0.922	3.00 (0.50)	3.20 (1.00)	0.101	0.666
STA	7.00 (4.00)	6.00 (1.00)	0.927	0.031	8.00 (6.00)	8.00 (9.00)	0.889	0.054

CA, cognitive anxiety; SA, somatic anxiety; SC, self-confidence; STA, state anxiety; n, number; IQR, interquartile range; *p*, *p*-value; *p < 0.05; *d*, Cohen’s d.

## Discussion

The aim of the present study was to evaluate anxiety and self-confidence prior to training matches and sports competition in high level men’s padel players from Finland. The initial hypothesis positing higher levels of anxiety and lower levels of self-confidence before competitive matches compared to training matches was only partially supported by the findings. Our findings revealed significant differences in self-confidence and somatic anxiety. Specifically, higher levels were reported prior to competition than before training matches. These observed differences may be attributed to several key factors. Firstly, the heightened excitement and importance associated with official matches might inspire top players to exhibit their utmost self-assurance. The challenges inherent in an official game may lead athletes to rely on their skills and positive beliefs, thereby boosting their self-confidence and somatic anxiety levels. Additionally, the structured and organized nature of competitive play, coupled with the presence of spectators and the pursuit of tangible outcomes, may foster a heightened sense of readiness and physiological arousal in players. In comparison to previous research, our findings align with certain patterns observed by Cervantes Blásquez et al. (2009) in swimmers, where similar differences in somatic anxiety were noted between training and competition. Likewise, Souza et al. (2019) found that somatic anxiety remained stable in canoe athletes, street runners and jiu-jitsu fighters a few days prior to the event but show a sudden rise and reach a peak at the onset of the competition. Interestingly, Mateo et al. (2012) also showed that somatic anxiety was lower 3 days before the first day of competition in BMX riders, although the opposite happened to self-confidence.

Another hypothesis was that left-side players, as well as right-side players, will show higher levels of anxiety and lower levels of self-confidence before competition than prior to training matches. This hypothesis was partially accepted. We observed that left-side players presented higher levels of somatic anxiety and self-confidence before competition than before training matches. However, it is crucial to note that no significant differences were found among right-side players in the same comparison, indicating unique psychological responses based on player positions. These results may be indicative of the distinctive responsibilities and pressures experienced by players during critical moments in a match. The higher involvement of the left-side player in the penultimate and last shots of the points (Ramón-Llín et al., 2022) aligns with the idea that their perceived role in shaping match outcomes could contribute to heightened somatic anxiety and self-confidence before competition.

In addition, it was hypothesized that both prior to training and competitive matches, left-side players will present lower levels of anxiety and higher levels of self-confidence when compared to right-side players. This was partially accepted, since left-side players only presented lower levels of somatic anxiety and state anxiety prior to training matches. As mentioned before, it is common in a padel pair consisting of right-handed players to position the best player on the left-side. This strategic choice is often made because it increases the likelihood of the player hitting more shots, especially overheads. It is worth highlighting that this is a pioneer study and future research should keep evaluating these trends.

It was also hypothesized that higher ranked players will show similar levels of anxiety and self-confidence between competition and training matches while lower ranked players will show lower levels of self-confidence and higher levels of anxiety before competition than training matches. The findings showed that among higher ranked players, somatic anxiety and self-confidence was higher prior to competition than before training matches. And, among lower ranked players, self-confidence was higher prior to competition than before training matches. The higher somatic anxiety observed among higher-ranked players before competition may be due to the fact that their performance is closely scrutinized, the stakes are higher, and there is an increased pressure to maintain their reputation and rankings, all of which contribute to a heightened arousal. This is consistent with findings from Cervantes Blásquez et al. (2009), who similarly observed higher somatic anxiety levels among athletes prior to competition compared to a simulated competition during training, as evidenced in their research with swimmers. For both the higher-and the lower-ranked players, the higher self-confidence before competition may be a result of the positive effects of pressure training. This is in accordance with Low et al. (2023), in which some international athletes were interviewed and admitted that their self-confidence was boosted due to the pressure training.

Additionally, it was hypothesized that match winning players will show similar levels of anxiety and self-confidence between competition and training while match losing players will show lower levels of self-confidence and higher levels of anxiety before competition than training matches. The results showed that there were significant differences only among winning players, in somatic anxiety and self-confidence. Specifically, these players presented higher values before competitive matches than before training matches. The pressure training conducted during the weeks leading up to the competition may have played a pivotal role in shaping the psychological preparedness of winning players (Gröpel and Mesagno, 2017; Kent et al., 2018). It is also worth noting that the positive effect of self-confidence on competitive success has been confirmed by several studies (Vealey and Greenleaf, 2001; Hassmén et al., 2004; Jekauc et al., 2023).

In a practical context, coaches are urged to engage in pressure training to enhance the readiness of their athletes for competitive situations (Stoker et al., 2016; Low et al., 2023). Similarly, it is recommended that athletes participate in psychological training to cultivate mental skills and effectively apply them under pressure (Lange-Smith et al., 2023). Moreover, coaches should consider psychological training to facilitate effective communication (Mora et al., 2009) with their players, conduct productive training sessions, and provide constructive feedback in both training and competitive environments.

This study possesses several notable strengths. Firstly, it marks the pioneering implementation of pressure training in the field of padel. Secondly, it stands as the inaugural research endeavor to examine precompetitive anxiety and self-confidence levels based on the side of play, as well as to compare precompetitive anxiety and self-confidence levels between players of the same side at the same moment (pre-training and pre-match, respectively). Thirdly, the implications drawn from these findings hold substantial practical value for coaches and sport psychologists. It is imperative to consider these results in designing effective pressure training programs for athletes.

It is essential to underscore certain limitations inherent in this investigation. For future investigations, the implementation of randomized controlled trials in both genders is recommended to more accurately assess the impact of pressure training. Additionally, incorporating alternative tools like pulsometers, which measure heart rate variability, alongside traditional questionnaires would provide a more comprehensive understanding of precompetitive anxiety and self-confidence. Furthermore, expanding the participant pool to include both elite and amateur-level players is advisable for a more nuanced exploration of the psychological effects of pressure training in different players. Moreover, exploring the effects of pressure training on padel performance could provide a holistic understanding of the impact of this intervention on actual gameplay. In addition, it would be necessary to include the influence of left-handed players in this analysis in future studies.

## Conclusion

The levels of anxiety and self-confidence before training matches and competitive matches have been described in high-level men’s padel players from Finland who underwent pressure training in the two leading weeks to a competition. The analysis, accounting for variables such as side of play, ranking and match outcome, reveals distinctive patterns.

Pressure training exerts a discernible impact on players, manifesting in lower self-confidence and similar levels of cognitive and state anxiety, juxtaposed with elevated somatic anxiety before training matches when compared to competitive matches. This trend persists across left-side, higher-ranked and match winning players. Intriguingly, even lower-ranked players display heightened self-confidence ahead of competitions.

These findings offer valuable insights for players, coaches, and sports psychologists, enriching their understanding of the intricate interplay between pressure training, competition, and the athlete’s psychological landscape. In practical terms, the results suggest that players can benefit from honing their mental skills and engaging in pressure training to optimize performance. Consequently, padel coaches are encouraged to consider psychological training, fostering effective support for their athletes. Hence, the incorporation of a sport psychologist within teams could prove instrumental in maximizing the psychological well-being and performance potential of padel players.

## Data availability statement

The raw data supporting the conclusions of this article will be made available by the authors, without undue reservation.

## Ethics statement

The studies involving humans were approved by Ethics committee of the Universidad Europea de Madrid (CIPI/22.303). The studies were conducted in accordance with the local legislation and institutional requirements. The participants provided their written informed consent to participate in this study.

## Author contributions

RC-R: Conceptualization, Data curation, Formal analysis, Investigation, Methodology, Resources, Validation, Writing – original draft, Writing – review & editing. AE-T: Supervision, Validation, Writing – original draft. ÁB-S: Conceptualization, Funding acquisition, Investigation, Methodology, Project administration, Supervision, Validation, Writing – original draft, Writing – review & editing.
